# The therapeutic effects of qigong in patients with chronic obstructive pulmonary disease in the stable stage: a meta-analysis

**DOI:** 10.1186/s12906-019-2639-9

**Published:** 2019-09-04

**Authors:** Hongxuan Tong, Yihua Liu, Yutian Zhu, Boli Zhang, Jingqing Hu

**Affiliations:** 1 Institute of Basic Theory for Chinese Medicine, China Academy of Chinese Medical Sciences, No. 16 South Street, Dongzhimen, Dongcheng District, Beijing, 100700 China; 2 Beijing University of Chinese Medicine, Beijing, 100029 China; 3 Department of Urology, Peking University Third Hospital, Beijing, 100191 China; 4 Department of Andrology, Peking University Third Hospital, Beijing, 100191 China; 5 Chinese Academy of Chinese Medical Sciences, Beijing, 100700 China; 6 Tianjin University of Chinese Medicine, Tianjin, 301617 China

**Keywords:** Qigong, Chronic obstructive pulmonary disease, Lung function, 6-minute walk distance

## Abstract

**Objectives:**

Chronic obstructive pulmonary disease (COPD) is one global disease. Lung function gradually declines. Medication does not fully reverse the airflow limitation. Qigong’s role in COPD rehabilitation has been assessed. We aimed to assess the effects of Qigong practised by COPD patients.

**Methods:**

Eligible articles were obtained through a systematic search. The databased were search on October 8, 2017, and the date range of the searches in the electronic databases had no upper limit. The Cochrane risk-of-bias tool was used to evaluate the quality of the eligible studies. Mean differences with 95% confidence intervals were utilized to analyse the results.

**Results:**

Ten included studies contained 993 participants. Statistical improvements occurred in the 6-min walk distance (6MWD) (MD, 30.57 m; 95% CI, 19.61–41.53 m; *P* < 0.00001); forced expiratory volume in 1 s (FEV1) (MD, 0.32 L; 95% CI, 0.09–0.56 L; *P <* 0.001); forced vital capacity rate of 1 s (FEV1/FVC) (MD, 2.66%; 95% CI, 1.32–2.26%; *P =* 0.0001); forced expiratory volume in 1 s/predicted (FEV1/pre) (MD, 6.04; CI, 2.58–9.5; *P =* 0.006); Monitored Functional Task Evaluation (MD, 0.88; 95% CI, 0.78–0.99; *P <* 0.00001); COPD Assessment Test for exercise (MD, − 5.54; 95% CI, − 9.49 to − 1.59; *P =* 0.006); Short Form-36 Health Quality Survey (SF-36)–General Health (MD, 5.22; 95% CI, 3.65–6.80; *P <* 0.00001); and Short Form-36 Health Quality Survey (SF-36)–Mental Health (MD, − 1.21; 95% CI, − 2.75 to 0.33; *P =* 0.12).

**Conclusions:**

In this meta-analysis of RCTs between ten included studies, we found that Qigong can improve COPD patients in lung function, exercise capacity and quality of life who were in the stable stage.

**Electronic supplementary material:**

The online version of this article (10.1186/s12906-019-2639-9) contains supplementary material, which is available to authorized users.

## Background

Chronic obstructive pulmonary disease (COPD) is one respiratory system disease the main characteristics of which are persistent respiratory tract symptoms and airflow limitation because of an abnormal airway and/or alveolae. [1] The prevalence of COPD is nearly 11.7% in the world, which means that it affects almost 400 million people, making it a common disease worldwide. [2]. It is the third deadly disease which is also one medical burden on patients and healthcare systems across the globe. [3–5] Symptoms such as dyspnoea, sputum, cough, gasping and difficulty breathing always occur in patients with COPD; lung function declines over time, and medication does not fully reverse the airflow limitation. [6, 7] In addition, the inflammation and/or the changes in repair mechanisms that are accompanied by the release of inflammation mediators may induce or aggravate comorbid diseases such as lung cancer, osteoporosis, cardiovascular disease, depression, anxiety, muscle weakness, and diabetes. [8, 9] It is therefore one common condition that is encountered by most COPD patients in primary care settings who need perpetual medical management. [10]

As a mind-body practice, Qigong combines meditation, respiratory regulation, and slow physical activity with or without visual imagery to harmonize the body, spirit, and mind. [11–13] It originates from Chinese history and philosophy, which dates back several centuries. [14] Qigong is an important practice in traditional Chinese medicine that aims to maintain physical health, psychological health, manage symptoms, and promote recovery. [15, 16] Qigong trains the body and mind to improve and recover the Qi; therefore, it is defined as “the art and science of refining and cultivating (*gong*) internal energy (*qi*), with the aim to encourage and accelerate the body’s ability to heal itself.” [14, 17–19] There are many styles of Qigong, such as Wuqinxi (“Five Animals Qigong”) and Baduanjin (“Eight Section Brocade Qigong”), Yijinjing, and Liuzijue. Previous studies have shown that Qigong exercise can benefit patients with multiple diseases such as cancer, [20] cardiovascular system disease, [12, 21] mental disease, [22, 23] and Parkinson’s disease, [24] and it promotes relaxation, reduces anxiety, reduces clinical somatic symptoms and stabilizes the sympathetic nervous system.

Practitioners of Traditional Chinese medicine also recommend Qigong to COPD patients to improve the function of internal organs, [25] and Qigong has been investigated to assess its effects in individuals with stable COPD. We conducted this review using a meta-analysis approach in which we undertook a comprehensive quantitative analysis to specifically assess the effectiveness of Qigong in stable COPD patients.

## Methods

### Literature search

Two investigators (*H. Tong* and Y. Liu) found eligible articles through a systematic search. They independently searched databases such as EMBASE, PubMed, Web of Science, Cochrane, WangFang data, China National Knowledge Infrastructure, and VIP Database for Chinese Technical Periodicals, without the upper-limit time until October 8, 2017, without any language restrictions. The following search terms were used: “Qigong”, “Ch’i Kung”, “Yindao”, “Gongfa”, “Baduanjin”, “Eight brocade”, “Yijinjing”, “Five Animals Exercise”, “Liuzijue”, “Six Character Formula”, “Daoyin yangsheng gong”, “Shierduanjin”, “12 brocade”, “Big dance”, “Mawangdui guidance”, “Tai Chi stick”, “Tai Chi staff”, “COPD” and “chronic obstructive pulmonary disease”. The detailed search strategy is in Additional file 1. The styles of Qigong that we finally selected were recommended by the Health Qigong Administrative Center of the General Administration of Sport of China (Beijing, China; details are in Additional file 2).

After removing repetitive articles, two investigators (*H. Tong* and Y. Liu) reviewed the abstracts independently. Studies were eliminated if they were reviews, meta-analyses, observational studies, case reports, case series, letters to the editor, comments, or irrelevant. Randomized controlled trials (RCTs) were included if the human participants were diagnosed as having stable COPD. [26] Studies were included if patients had participated in Qigong exercises longer than 6 months after diagnosis. However, trials were excluded if Qigong was combined with other similar energy practices, such as yoga techniques and meditation. Meanwhile, the control group only received conventional therapy such as routine health guidance and/or drug treatments. [27] Then, we searched the full articles for further assessment; articles without full text versions available were excluded. Any disagreements between the two investigators (*H. Tong* and Y. Liu) would refer to the third-party member’s opinion (Y. Zhu).

### Data extraction and analysis

Two investigators (*H. Tong* and Y. Liu) extracted general information from the aforementioned selected publications. Disagreements were resolved by a third investigator (Y. Zhu). The information of each eligible study contained the article name, first author, journal name, year of publication, group information, participating centres, country, observational sites, intervention duration, sample size, final statistics, ratio of male to female participants, study design, control group and average age.

The primary outcomes used to evaluate the effect of Qigong were the 6MWD, FEV1, FEV1/FVC, FEV1/pre, Monitored Functional Task Evaluation, COPD Assessment Test for exercise, Short Form-36 Health Quality Survey (SF-36) for General Health, and SF-36 for Mental Health. We emailed authors to request any missing data needed for our analysis. The differences between the control and experimental groups were evaluated by the effect sizes and 95% confidence intervals (CIs), which were calculated in Review Manager (version 5.3). As to continuous data, mean differences (MDs) were estimated. The *I*^2^ statistics reflected heterogeneity as follows: *I*^2^ = 0% indicated no heterogeneity; *I*^2^ = 0–25% indicated low heterogeneity; *I*^2^ = 25–50% indicated mild heterogeneity; *I*^2^ = 50–75% indicated moderate heterogeneity; and *I*^2^ = 75–100% indicated high heterogeneity. [28] Funnel plot would be used to reflect the potential publication bias if there were more than ten studies.

### Quality and risk-of-bias assessments

We used the Cochrane Handbook for Systematic Reviews of Interventions to assess the quality and risk of bias for each selected article. Two authors (*H. Tong* and Y. Liu) reviewed all selected articles and reported their evaluations as “high,” “low,” or “unclear” for the following items: (1) selection bias; (2) blinding; (3) attrition bias; (4) reporting bias; (5) other biases. [29] To improve accuracy, any disagreements would refer to a third member’s opinion. The quality and risk-of-bias assessments were described in Additional file 3.

## Results

### Study search

**Fig. 1:**
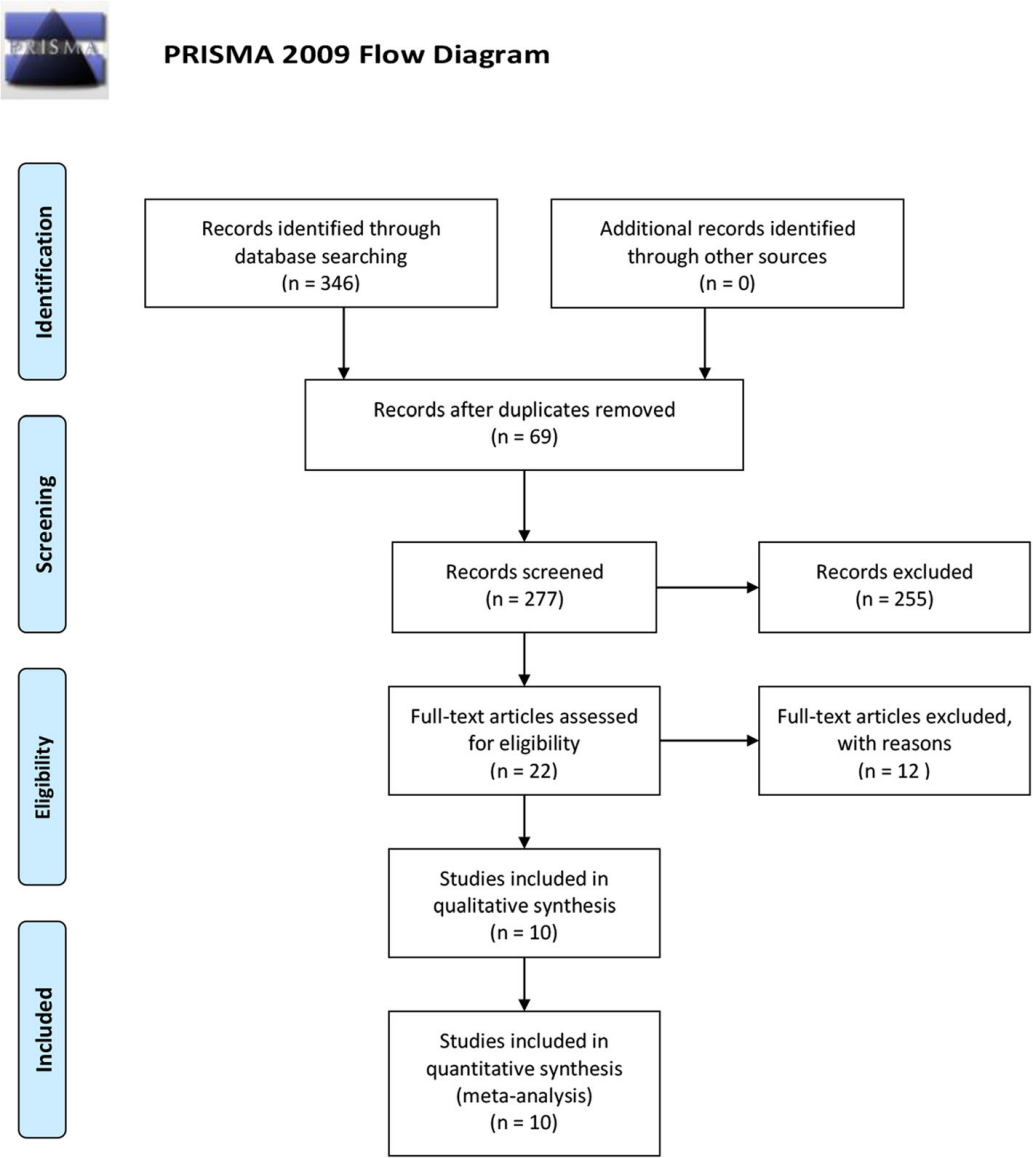
Search strategy and flow chart of the screened, excluded, and analysed articles

Figure 1 shows the flow diagram of the selection process. All databases yielded 346 potentially relevant articles. Sixty-nine duplicate studies were eliminated. After screening the abstracts, 255 studies were excluded based on their titles and summaries. There were 22 eligible studies for full-text screening; 10 studies were subsequently selected for the final analysis. Details of the excluded articles are provided in Additional file 4.

Among the 10 studies [25, 30–38] selected for this meta-analysis, four studies [25, 30–32] were published in English, and six studies [33–38] were published in Chinese, two of which were dissertations. [37, 38] The period in which these studies were published was from 2010 to 2016. The number of participants ranged from 22 individuals to 148 individuals, and the total number of participants was 993 individuals. The Qigong exercises used were Baduanjin in five studies, [25, 33, 35–37] Liuzijue in two studies, [31, 34] and Yijinjing in two studies. [32, 38] One study [30] used reproduced Qigong derived from the aforementioned three types of Qigong and Wuqinxi. More details are provided in Table 1.

**Table 1:** The detailed characteristics of each selected study

Author	Study design	Initial inclusion of patients	Mean age, years (I/C)	Intervention in the control group	Intervention in the Qigong group	Actual number in control group	Actual number in experimental group	Qigong style	Protocol for Qigong
Xu 2010 [33]	RCT	40	57.02/57.51	Conventional drugs	Conventional drugs + Qigong	20	20	Baduanjin	60 min/time, 1 time/day, 1 year
Xue 2015 [35]	RCT	59	66.73/63.89	Conventional drugs	Conventional drugs + Qigong	28	31	Baduanjin	30 min/time, at least 4 times/week, 6 months
Guo 2016 [36]	RCT	120	70.87/70.79	Conventional drugs + routine health advice + walking	Conventional drugs + routine health advice + Qigong	56	55	Baduanjin	30 min/time, 4 times/week, 36 weeks
Booby 2011 [25]	RCT	80	71.7/73.1	Conventional rehabilitation + walking	Qigong	29	23	Baduanjin	45 min/time, at least 4 times/week,6 months
Yi 2013 [37]	RCT	22	70.40/69.17	Conventional drugs + breathing exercise	Conventional drugs + Qigong	12	10	Baduanjin	30 min/time, 1 time/day, 6 months
Wang 2013 [34]	RCT	87	71.16/74.40	Conventional drugs + conventional rehabilitation	Conventional drugs + conventional rehabilitation + Qigong	41	39	Liuzijue	30 min/time, 2 times/day, 1 year
Xiao 2015 [31]	RCT	126	72.20/70.90	Walking	Walking + Qigong	60	59	Liuzijue	45 min/time, at least 4 times/week, 6 months
Gao 2015 [38]	RCT	120	71.42/74.24	Conventional drugs + conventional rehabilitation	Conventional drugs + conventional rehabilitation + Qigong	57	55	Yijinjing	60 min/time, 2 times/day, 6 months
Zhang 2016 [32]	RCT	148	64.77/62.35	Conventional rehabilitation	Conventional rehabilitation + Qigong	45	42	Yijinjing	60 min/day, split the 60-min practice time into morning and afternoon sessions, 6 months
Liu 2012 [30]	RCT	96	61.82/62.20	Conventional rehabilitation	Conventional rehabilitation + Qigong	35	51	Reproduced Qigong	60 min/time, 3 times/week, 6 months

*I/C* intervention/control, *RCT* randomized controlled trial

### Six-minute walk distance

Eight [25, 30–33, 36–38] of the 10 trials used the 6MWD test to evaluate the effect of Qigong and compared a patient group with a control group. One trial [36] chose 24 weeks and 36 weeks as the observation time points. We chose the 36-week timepoint because 24 weeks is slightly less than 6 months. We used the different types of Qigong to establish subgroups in our analysis.

**Fig. 2:**
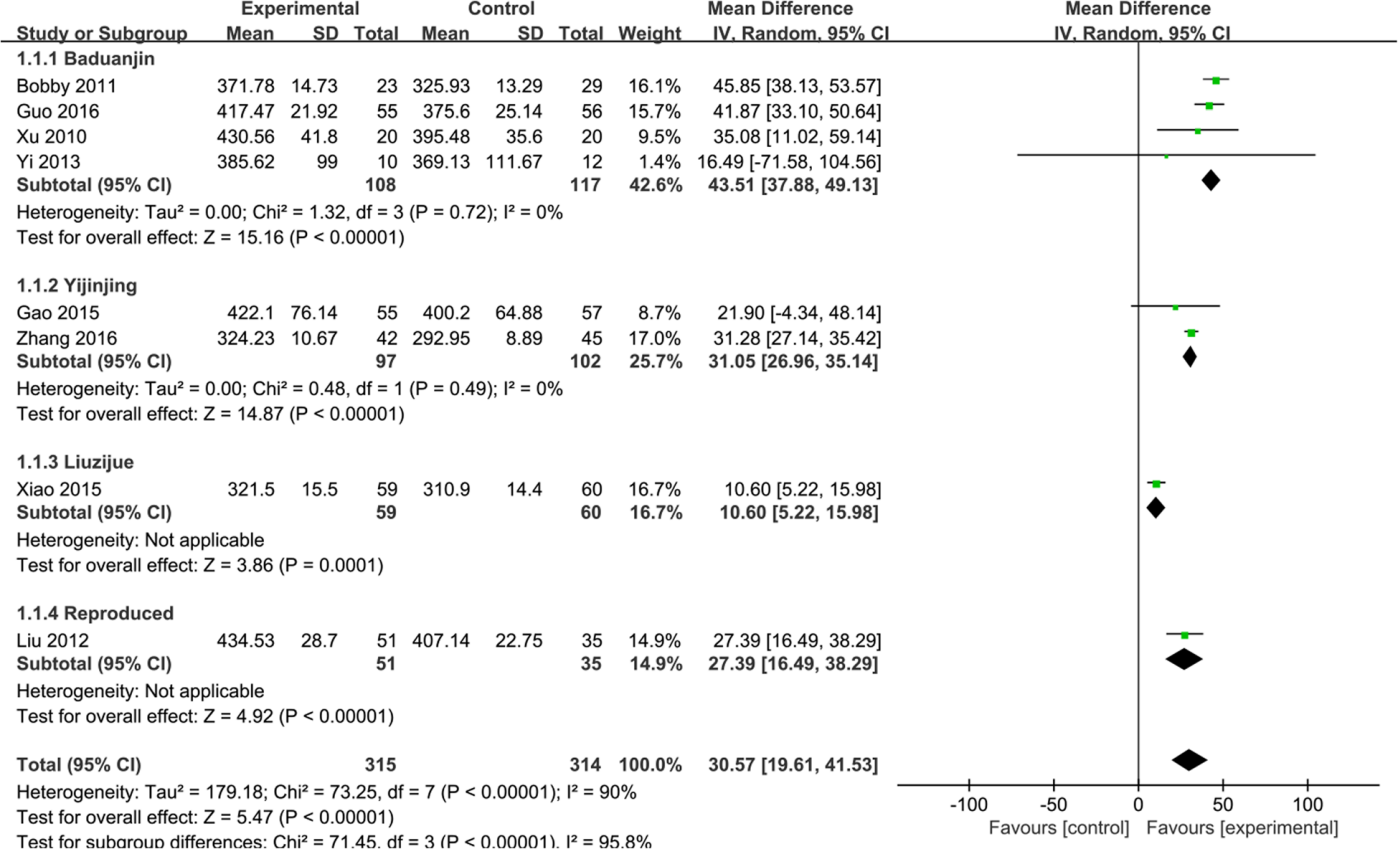
Effects of Qigong on the 6-min walk distance (6MWD). Subgroup meta-analysis was used to assess the effects of different types of Qigong on the 6MWD with a fixed effects model

The random effects analysis was conducted to merge the results because eight studies [25, 30–33, 36–38] had high heterogeneity (*I*^2^ = 90%). The effect size of the eight studies found that Qigong was associated with an improvement in 6MWD test results significantly compared to the control intervention (MD, 30.57 m; 95% CI, 19.61–41.53 m; *P <* 0.00001; Fig. 2). Baduanjin resulted in the best improvement in the 6MWD (MD, 43.51 m; 95% CI, 37.88–49.13 m; *P <* 0.00001; Fig. 2). The other three exercise types also resulted in significant improvements in the 6MWD: Yijinjing (MD, 31.05 m; 95% CI, 26.96–35.14 m; *P <* 0.00001); Liuzijue (MD, 10.60 m; 95% CI, 5.33–15.98 m; *P =* 0.0001); and reproduced Qigong (MD, 27.39 m; 95% CI, 16.49–38.29 m; *P <* 0.00001).

### Lung function

**Fig. 3:**
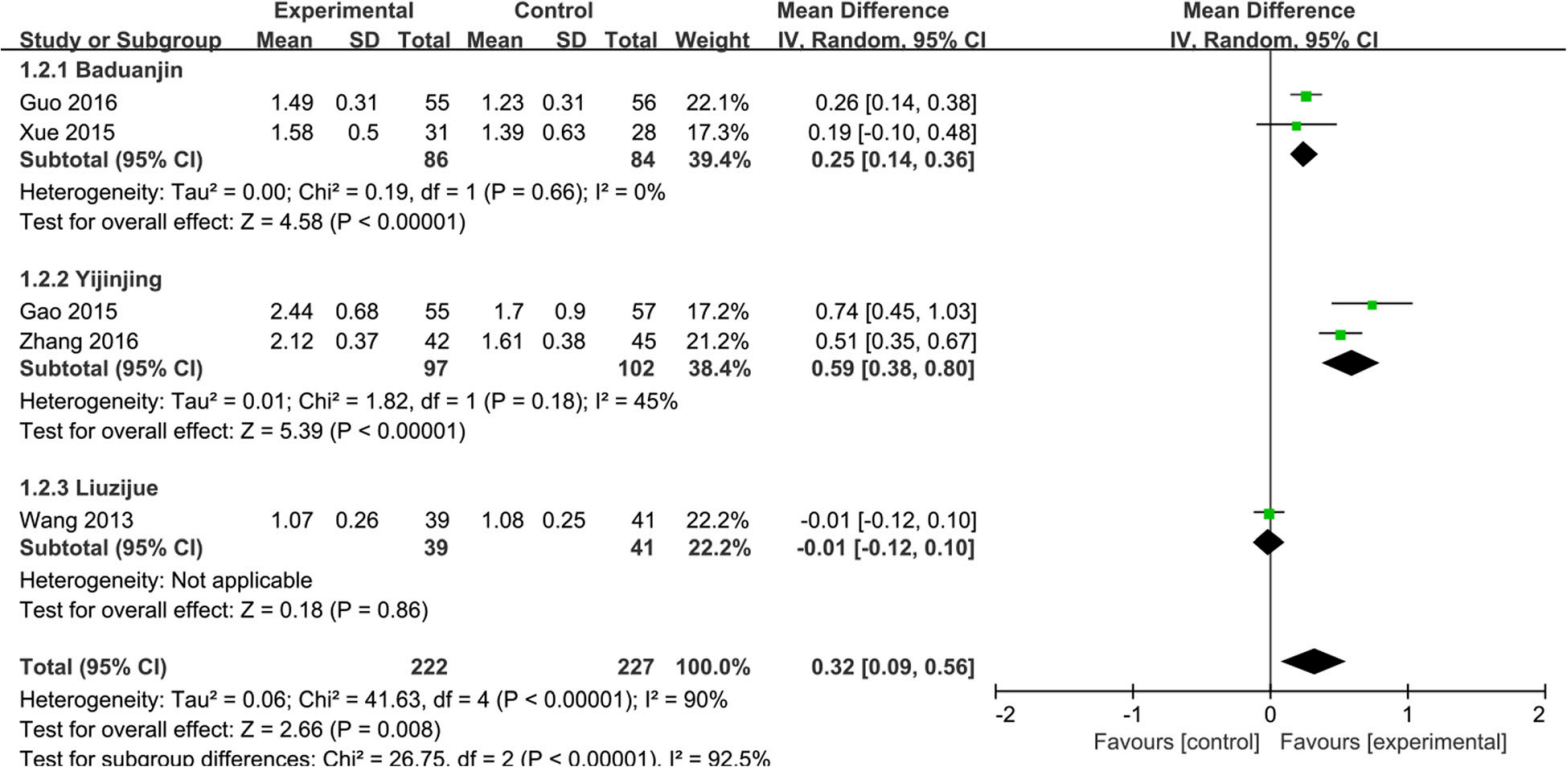
Effects of Qigong on forced expiratory volume in 1 s (FEV1). Subgroup meta-analysis was used to assess the effects of different types of Qigong on FEV1 with a random effects model

**Fig. 4:**
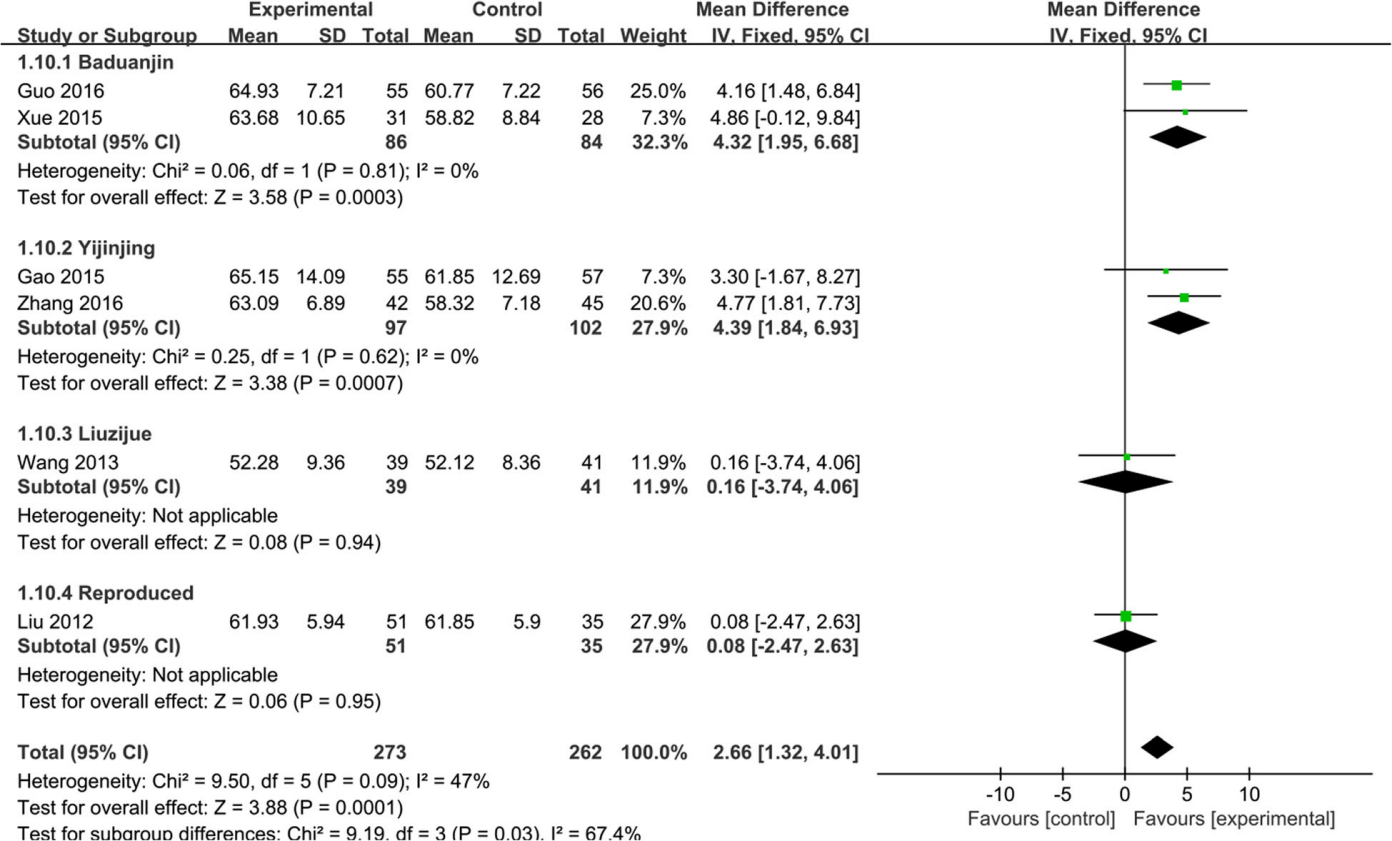
Effects of Qigong on the forced vital capacity rate of 1 s/forced vital capacity (FEV1/FVC). Subgroup meta-analysis was used to assess the effects of different types of Qigong on the FEV1/FVC with a fixed effects model

**Fig. 5:**
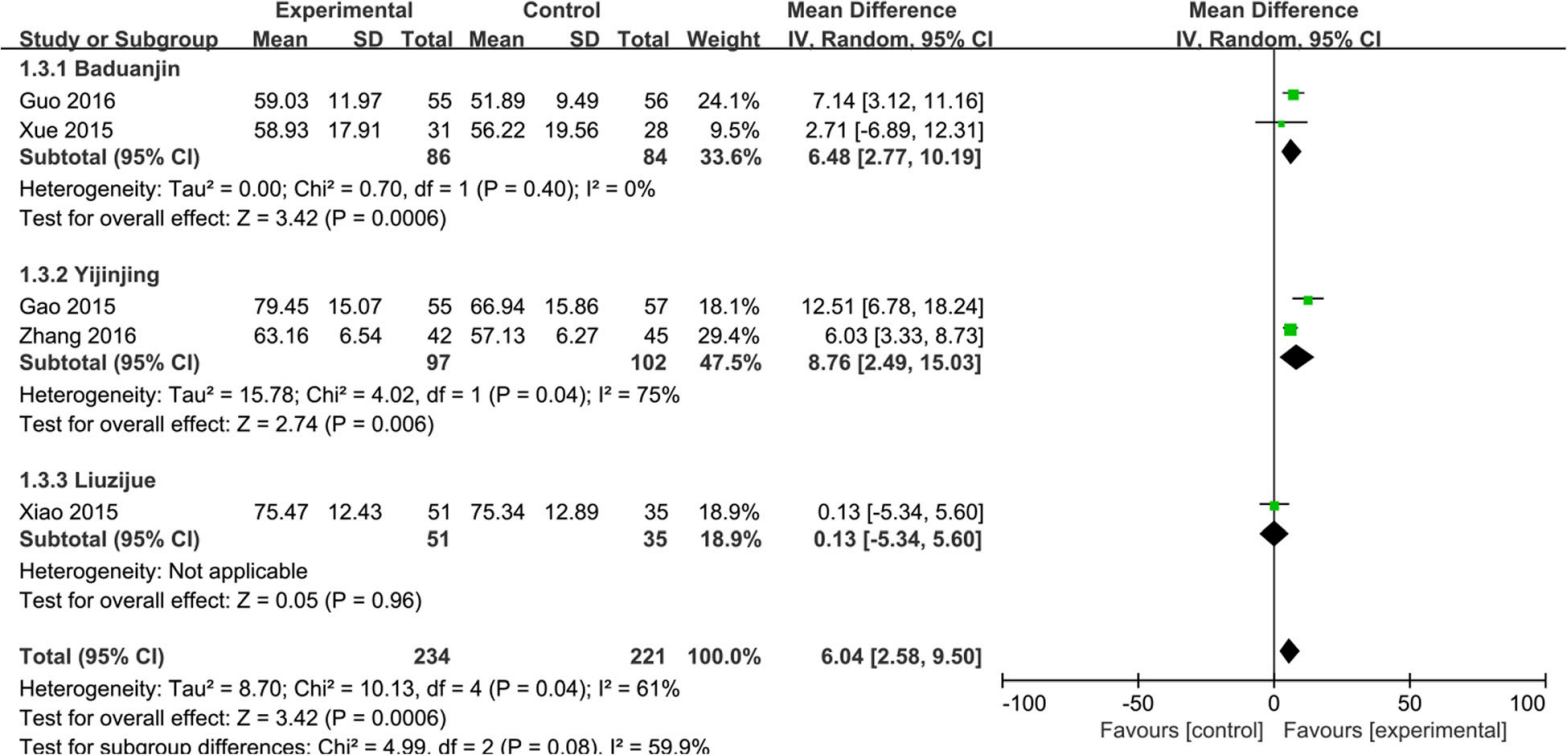
Effects of Qigong on the forced vital capacity rate of 1 s (FEV1/pre). Subgroup meta-analysis was used to assess the effects of different types of Qigong on the FEV1/pre with a random effects model

Five [32, 34–36, 38] of the 10 trials used FEV1 to evaluate the effect of Qigong in COPD patients. The random effects analysis was conducted to incorporate results because the combined trials had high heterogeneity (*I*^2^ = 90%). We used the different styles of Qigong to establish subgroups. The effect size of the five trials [32, 34–36, 38] showed that Qigong could significantly improve the FEV1 (MD, 0.32; 95% CI, 0.09–0.56; *P* < 0.001; Fig. 3). Yijinjing, which was used in 2 trials, [32, 38] resulted in the best improvement in the FEV1 (MD, 0.59; 95% CI, 0.38–0.80; *P* < 0.00001; Fig. 3). Baduanjin also had a good effect (MD, 0.25; 95% CI, 0.14–0.36; *P* < 0.00001; Fig. 3). One trial, [34], which used Liuzijue, did not show a positive effect. Six [30, 32, 34–36, 38] of the 10 trials used the FEV1/FVC to assess the improvement in lung function. A fixed effects analysis was conducted to incorporate the results (*I*^2^ = 47%). The effect size of the six trials [30, 32, 34–36, 38] showed that Qigong could significantly improve the FEV1/FVC (MD, 2.66; 95% CI, 1.32–2.26; *P* = 0.0001; Fig. 4). Yijinjing showed a treatment effect (MD, 4.39; 95% CI, 1.84–6.93; *P* = 0.0007; Fig. 4), and Baduanjin also improved the FEV1/FVC (MD, 4.32; 95% CI, 1.95–6.68; *P* = 0.0003; Fig. 4). However, Liuzijue and reproduced Qigong did not have significant effects. Five trials [31, 32, 35, 36, 38] used the FEV1/pre to assess the improvement in lung function (MD, 6.04 CI, 2.58–9.5; *P* = 0.006; Fig. 5). The random effects analysis was conducted to pool the results together (*I*^2^ = 61%). Yijinjing showed the best results (MD, 8.76; 95% CI, 2.49–15.03; *P =* 0.006; Fig. 5). Baduanjin resulted in a significant improvement (MD, 6.48; 95% CI, 2.77–10.19; *P =* 0.0006; Fig. 5), but Liuzijue did not result in a significant improvement (MD, 0.13; 95% CI, − 5.34 to 5.60; *P =* 0.96; Fig. 5).

### Other evaluation indexes

**Fig. 6:**
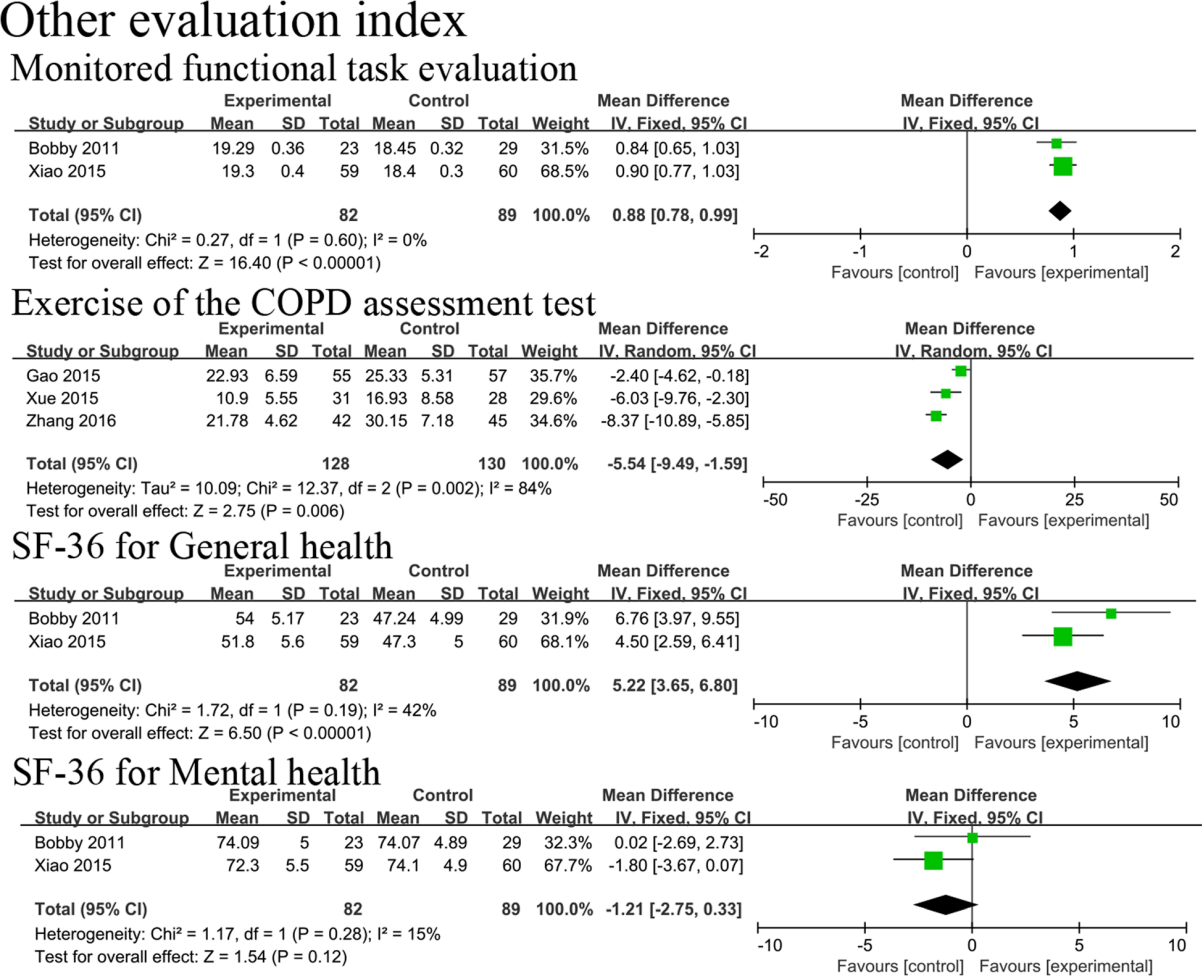
Effects of Qigong on the results of the Monitored Functional Task Evaluation, COPD Assessment Test for exercise, SF-36 for General Health, and SF-36 for Mental Health SF-36, Short Form-36 Health Quality Survey.

Two [25, 31] of the 10 studies used the Monitored Functional Task Evaluation to assess the activities of daily living for COPD patients in the stable stage. The effect of the two trials showed that Qigong resulted in a significant improvement in the Monitored Functional Task Evaluation results compared to the control group (MD, 0.88; 95% CI, 0.78–0.99; *P <* 0.00001; Fig. 6). There were three trials [32, 35, 38] that used the COPD Assessment Test for exercise to evaluate the severity of COPD. This assessment revealed that Qigong significantly improved the COPD assessment test results for exercise in COPD patients (MD, − 5.54; 95% CI, − 9.49 to − 1.59; *P =* 0.006; Fig. 6). Two trials [25, 31] used the SF-36 for General Health and the SF-36 for Mental Health to evaluate physical and psychological health, respectively. The general health results showed that Qigong resulted in a significant improvement for COPD patients (MD, 5.22; 95% CI, 3.65–6.80; *P <* 0.00001; Fig. 6). However, there was no statistic difference in mental health (MD, − 1.21; 95% CI, − 2.75 to 0.33; *P =* 0.12; Fig. 6). We did not conduct subgroup analyses in this study because of the small number of trials.

## Discussion

COPD rehabilitation is a key treatment for COPD patients advised by international and national guidelines, and the recommended practices for rehabilitation are physical exercise, patient-oriented education, and smoking cessation. [39–42] As an important supplement of physical exercise in COPD patients, Qigong achieves physical and mental integration through specific actions, breathing techniques, and meditation, which can regulate the patient’s energy (*qi*) to benefit the patient’s physical, psychological, and spiritual health. [43] We conducted this meta-analysis to evaluate the curative effects of Qigong (e.g., Baduanjin, Yijinjing, Liuzijue, and reproduced Qigong) in patients with stable COPD. Here, we only focused on Qigong exercise, which differed from the former research studies that assessed the effectiveness of Tai Chi and Qigong at the same time or did not separate these different styles of exercise as subgroups in their analyses. [27, 44, 45]

In our study, we created subgroups based on the different styles of Qigong to assess the total effect and each style’s effect, which would provide COPD patients with a better means of making an informed choice when they are presented with so many exercises. In general, compared with conventional treatment, our study found that Qigong exercises could promote COPD rehabilitation as assessed by the 6MWD, FEV1, FEV1/FVC, FEV1/pre, Functional Task Evaluation, SF-36 for General Health, and COPD Assessment Test for exercise in stable COPD patients. However, there was no significant improvement in the SF-36 for Mental Health.

The 6MWD is a clinical exercise endurance evaluation index that can reflect a patient’s functional status. In our study, we found that the length of the 6MWD in the Qigong groups was increased compared with the length in the control group. This finding represents an effective improvement in the patients’ exercise capacity, far exceeding the previously reported minimum clinically important differences. [46] In COPD patients, muscle strain is very common, and their lower limbs are weaker than those of healthy people; therefore, their athletic ability is gradually reduced. [47] Qigong contains specific lower limb movements that are features of the four styles of Qigong that can enhance the strength of the lower limbs and prolong the 6MWD.

Patients with COPD have decreased exercise capacity and increased dyspnoea during the progression of the disease because of lung function insufficiency. Adequate Qigong training could contribute to the improvement of health outcomes. In our study, Qigong improved lung function (i.e., FEV1, FEV1/FVC, and FEV1/pre), which reflected airway obstruction and disease severity. The findings in the subgroup analysis suggested that Baduanjin and Yijinjing could significantly improve lung function, whereas studies focusing on Liuzijue and reproduced Qigong found that these practices did not significantly improve lung function. This finding may partially be because Baduanjin and Yijinjing can increase the strength of the respiratory muscles, reduce the pulmonary residual volume, promote efficiency in gas exchange, and slow the decrease in lung function. [25, 35, 36, 48, 49] With regard to Liuzijue in the single clinical trial, it did not show a significant improvement in these functions, perhaps because it lacks sufficient upper limb movement, compared with Baduanjin and Yijinjing. [31] With regard to reproduced Qigong, the continuity of movement may be lost when selecting movements from Yijinjing, Wuqinxi, Liuzijue, and Baduanjin. Nevertheless, these practices could improve immune function and have a better protective effect against acute exacerbation of COPD, [30, 31] which would be beneficial for patients with COPD. These different improvements would give patients with COPD (exacerbation or stable stage) a better means of making an informed choice when they want to participate in a Qigong exercise programme.

The aim of respiratory rehabilitation is to improve lung function in COPD patients through Qigong exercises. We also believe that quality of life is an important outcome for these patients. We focused on a multiple evaluation index to assess the beneficial result of Qigong exercises. In this study, we used the subscales for general health and mental health in the Short Form-36 Health Quality Survey (SF-36). The COPD Assessment Test for exercise is a validated tool to measure the influence or burden of COPD on an individual. [50]

The practice of Qigong by patients with stable COPD can improve the results of the Functional Task Evaluation, SF-36 for General Health, and the COPD Assessment Test for exercise compared with conventional treatment. The actions in Qigong can train the upper limb and lower limb muscles and improve athletic ability, thereby reducing the burden on patients with COPD. In addition, Qigong can regulate immune responses to enforce the body’s natural self-healing ability so that patients do not become sick as easily and can recover faster. [25, 31] As a form of exercise, Qigong resulted in no significant difference in the SF-36 for Mental Health before and after the programme. However, this factor will still require more clinical trials to assess whether it has a psychological regulatory function.

### The limitations of our study

There were likewise some limitations in our meta-analysis. First, although there were 10 RCTs involving 993 individuals selected by us, the sample size of each study was relatively small. Meanwhile, all involved studies were conducted in China, and clinical studies should also be conducted in Western countries to evaluate the effect of Qigong, especially in different ethnicities. Moreover, we searched eight databases but did not search for unpublished trials. Finally, there was apparent heterogeneity because of the differences in the four selected types of Qigong, and we then used the types to generate subgroups in addition to analysing them collectively; not all studies used the same test index, and some of them only had one group in subgroup analysis. Therefore, it is necessary to update the data in the future when there have been more RCTs conducted.

## Conclusions

In our meta-analysis, we found that over a period of 6 months, Qigong resulted in an improvement in lung function, exercise capacity, and patients’ quality of life. Meanwhile, different styles of Qigong could have different benefits in stable COPD patients.

## Additional files

Additional file 1: Detailed search strategy. The detailed search strategies for EMBASE, PubMed, Web of Science, Cochrane, China National Knowledge Infrastructure, WangFang, and VIP Database for Chinese Technical Periodicals. (DOCX 18 kb)

Additional file 2: Quality and risk of bias assessments. The risk of bias graph and risk of bias summary assessed according to the Cochrane handbook. Risk of bias graph: review authors’ judgements about each risk of bias item presented as percentages across all included studies. Risk of bias summary: review authors’ judgements about each risk of bias item for each included study. (TIF 1852 kb)

Additional file 3: Details of excluded articles. The excluded articles in the stage of full text assessment. (XLS 25 kb)

Additional file 4: The support information about the different types of Qigong that were selected in our research. The support information about the different types of Qigong that were selected in our research as advised by the Health Qigong Administrative Center of the General Administration of Sport of China. (DOCX 15 kb)

## Data Availability

The data analysed and materials used in this study are available from the corresponding author on reasonable request.

## Abbreviations

CI: Confidence interval
COPD: Chronic obstructive pulmonary disease
FVC: Forced vital capacity
MD: Mean difference
MFTE: Monitored Functional Task Evaluation
RCT: Randomized controlled trial
