# Supplementary material for: The therapeutic effects of qigong in patients with chronic obstructive pulmonary disease in the stable stage: a meta-analysis
Source: BMC Complement Altern Med. 2019 Sep 4;19:239. doi: 10.1186/s12906-019-2639-9 (PMC6727520; doi:10.1186/s12906-019-2639-9)
Supplement: Supplementary file 1 — Detailed search strategy. The detailed search strategies for EMBASE, PubMed, Web of Science, Cochrane, China National Knowledge Infrastructure, WangFang, and VIP Database for Chinese Technical Periodicals. (DOCX 18 kb) [file 12906_2019_2639_MOESM1_ESM.docx]

**Search strategy：**

**Pubmed：**

#1 ("Qigong"[Mesh]) or (Qi Gong) or (Ch'i Kung) or (yindao) or (gongfa) or (Baduanjin) or (Eight * brocade) or (Yijin*Jing) or (five*animals exercise) or (Liuzijue) or (Six*character formula) or (DAOYIN YANGSHENG GONG) or (shierduanjin) or (Twelve*brocade) or (Big dance) or (Mawangdui guidance) or (Tai Chi*stick) or (Tai Chi*staff)

#2 ("Pulmonary Disease, Chronic Obstructive"[Mesh]) or (COPD) or (Chronic Obstructive Pulmonary Disease) or (COAD) or (Chronic Obstructive Airway Disease) or (Chronic Obstructive Lung Disease) or (Airflow Obstruction, Chronic) or (Airflow Obstructions, Chronic) or (Chronic Airflow Obstructions) or (Chronic Airflow Obstruction)

#3 (Humans[Mesh] AND (Clinical Trial[ptyp] OR Meta-Analysis[ptyp] OR Randomized Controlled Trial[ptyp] OR Comparative Study[ptyp] OR Controlled Clinical Trial[ptyp] OR "cohort"[All Fields] OR "evaluation"[All Fields]))

#4 #1 and #2

#5 #4 and #3

**Embase:**

#1 'qigong'/exp OR 'qi gong' OR yindao OR gongfa OR baduanjin OR (eight brocade) OR yijinjing OR (five animals exercise) OR liuzijue OR (six character formula) OR (daoyin yangsheng gong) OR shierduanjin OR (twelve brocade) OR (big dance) OR (mawangdui guidance) OR (tai chi stick) OR (tai chi staff)

#2 'chronic obstructive lung disease'/exp and 'chronic obstructive lung disease'

#3 #1 and #2 AND [humans]/lim

**Cochrane**

#1 MeSH descriptor: [Qigong] explode all trees

#2 MeSH descriptor: [Pulmonary Disease, Chronic Obstructive] explode all trees

#3 (Qi Gong) or (Ch'i Kung) or (yindao) or (gongfa) or (Baduanjin) or (Eight brocade) or (YijinJing) or (five animals exercise) or (Liuzijue) or (Six character formula) or (DAOYIN YANGSHENG GONG) or (shierduanjin) or (Twelve brocade) or (Big dance) or (Mawangdui guidance) or (Tai Chi stick) or (Tai Chi staff)

#4 #1 or #3

#5 #4 and #2

**Web of science**

#1 (TS=Qigong or TS=Ch'i Kung or TS=yindao or TS=gongfa or TS=Baduanjin or TS=Eight brocade or TS=five animals exercise or TS=Liuzijue or TS=YijinJing or TS=Six character formula or TS=DAOYIN YANGSHENG GONG or TS=shierduanjin or TS=Twelve brocade or TS=Big dance or TS=Mawangdui guidance or TS=Tai Chi stick or TS=Tai Chi staff)

#2 TS= Chronic Obstructive Pulmonary Disease or TS= COPD or TS= chronic obstructive lung disease

#3 #2 AND #1

**CNKI：**

(SU='气功' OR SU='引导' OR SU='功法' OR SU='八段锦' OR SU='易筋经' OR SU='五禽戏' OR SU='六字诀' OR SU='导引养生功' OR SU='十二段锦' OR SU='大舞' OR SU='马王堆导引术' OR SU='太极养生杖') and (SU='慢性阻塞性肺疾病' OR SU='COPD' OR SU='慢性阻塞性肺病' OR SU='慢阻肺' OR SU='慢性阻塞性肺气肿') and (SU='对照试验' OR SU='随机试验' OR SU='临床试验' OR SU='临床研究' OR SU='对照研究')

**Wangfang：**

(主题:(气功) or 主题:(引导) or主题:(功法) or 主题:(八段锦) or 主题:(易筋经) or 主题:(五禽戏) or 主题:(六字诀) or 主题:(导引养生功) or 主题:(十二段锦) or 主题:(大舞) or 主题:(马王堆导引术) or 主题:(太极养生杖)) and (主题:(慢性阻塞性肺疾病) OR 主题:(COPD) OR 主题:(慢性阻塞性肺病) OR 主题:(慢阻肺) OR 主题:(慢性阻塞性肺气肿)) and (主题:(对照试验) OR 主题:(随机试验) OR 主题:(临床试验) OR 主题:(临床研究) or 主题:(对照研究))

**VIP：**

((M=(气功+引导+功法+八段锦+易筋经+五禽戏+六字诀+导引养生功+十二段锦+大舞+马王堆导引术+太极养生杖))*(M=(慢性阻塞性肺疾病+COPD+慢性阻塞性肺病+慢阻肺+慢性阻塞性肺气肿))*(M=(对照试验+随机试验+临床试验+临床研究+对照研究)))+ ((R=(气功+引导+功法+八段锦+易筋经+五禽戏+六字诀+导引养生功+十二段锦+大舞+马王堆导引术+太极养生杖))*(R=(慢性阻塞性肺疾病+COPD+慢性阻塞性肺病+慢阻肺+慢性阻塞性肺气肿))*(R=(对照试验+随机试验+临床试验+临床研究+对照研究)))
