# Supplementary figures and images for: The therapeutic effects of qigong in patients with chronic obstructive pulmonary disease in the stable stage: a meta-analysis
Source: BMC Complement Altern Med. 2019 Sep 4;19:239. doi: 10.1186/s12906-019-2639-9 (PMC6727520; doi:10.1186/s12906-019-2639-9)

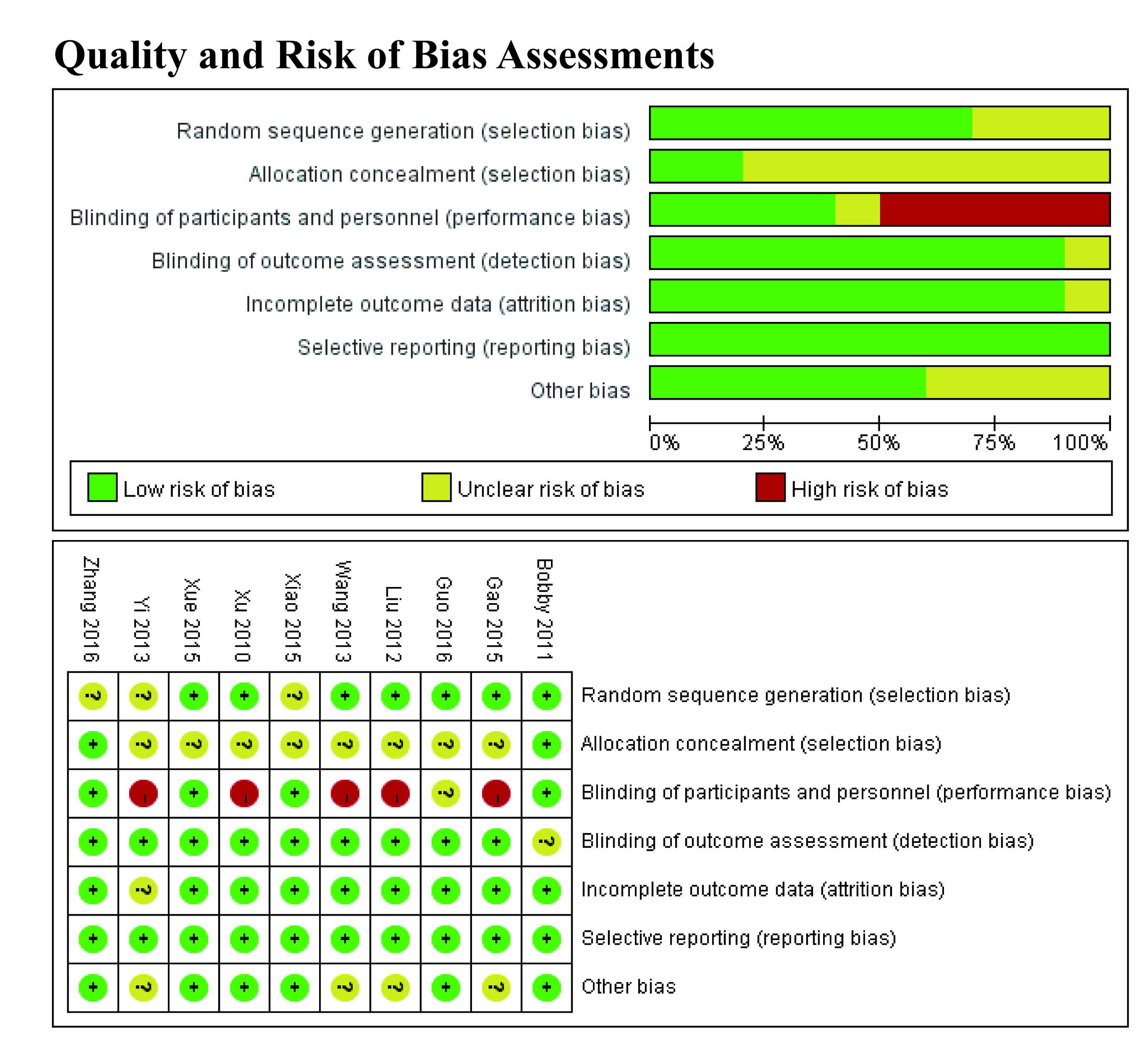

Supplement: Supplementary file 2 — Quality and risk of bias assessments. The risk of bias graph and risk of bias summary assessed according to the Cochrane handbook. Risk of bias graph: review authors’ judgements about each risk of bias item presented as percentages across all included studies. Risk of bias summary: review authors’ judgements about each risk of bias item for each included study. (TIF 1852 kb) [file 12906_2019_2639_MOESM2_ESM.tif]
