# Supplementary material for: The therapeutic effects of qigong in patients with chronic obstructive pulmonary disease in the stable stage: a meta-analysis
Source: BMC Complement Altern Med. 2019 Sep 4;19:239. doi: 10.1186/s12906-019-2639-9 (PMC6727520; doi:10.1186/s12906-019-2639-9)
Supplement: Supplementary file 4 — The support information about the different types of Qigong that were selected in our research. The support information about the different types of Qigong that were selected in our research as advised by the Health Qigong Administrative Center of the General Administration of Sport of China. (DOCX 15 kb) [file 12906_2019_2639_MOESM4_ESM.docx]

**Supplement 4**

The support information about the Qigong in our research advised by Health Qigong Administrative Center of the General Administration of Sport of China

Five animals exercise**:**

http://qgzx.sport.gov.cn/n5407/c670050/content.html; http://qgzx.sport.gov.cn/n5407/c781296/content.html

DaoYin YangSheng Gong:

http://qgzx.sport.gov.cn/n5407/c775167/content.html; http://qgzx.sport.gov.cn/n5407/c781303/content.html

Liuzijue:

http://qgzx.sport.gov.cn/n5407/c781307/content.html; http://qgzx.sport.gov.cn/n5407/c781291/content.html

Mawangdui guidance:

http://qgzx.sport.gov.cn/n5407/c781290/content.html

Big dance:

http://qgzx.sport.gov.cn/n5407/c781294/content.html

YijinJing:

http://qgzx.sport.gov.cn/n5407/c781295/content.html

Baduanjin:

http://qgzx.sport.gov.cn/n5407/c781297/content.html

Hangzhou sports bureau：

total：http://hzty.gov.cn/n11/n20/c3373/content.html
